# Physicochemical Properties of Avocado Seed Extract Model Beverages and Baked Products Incorporated with Avocado Seed Powder

**DOI:** 10.1155/2023/6860806

**Published:** 2023-05-31

**Authors:** Clinton O. Nyakang'i, Eunice Marete, Rebecca Ebere, Joshua M. Arimi

**Affiliations:** ^1^Department of Food Science, Meru University of Science and Technology, Meru, Kenya; ^2^Department of Physical Sciences, Meru University of Science and Technology, Meru, Kenya

## Abstract

Consumption of avocado (*Persea americana* Mill.) has increased worldwide in recent years. The avocado pulp is used, but the peel and seed are discarded as waste. Studies have shown that the seeds are rich in phytochemicals that can be utilized in food systems. The objective of this study was to evaluate the potential of Hass avocado seed as a source of polyphenols in the processing of model beverages and baked products with functional properties. The proximate analysis of the avocado seed powder was carried out. The shelf life of phenols in avocado seed powder (ASP) stored in dark, amber-colored bottle and transparent bottle was studied for six months. The seed extract was incorporated into model beverages of different pHs stored at refrigerated and ambient temperatures, and the shelf life was monitored for 20 weeks. The seed powder was incorporated into baked products at 0, 15, 30, or 50% followed by total phenolic content and sensory property analysis. Proximate composition of the seed powder for moisture, ash, protein, fiber, fat, and total carbohydrates was 14.19, 1.82, 7.05, 4.00, 13.64, and 59.30 percent, respectively. During storage of the seed powder, there was no significant difference (*P* > 0.05) in the phenol content under the different storage light conditions for six months. In the model beverages, lower pH levels (2.8, 3.8, and 4.8) and those stored at ambient temperatures (25°C) recorded lower phenol content than the control pH, i.e., 5.5, and those under refrigerated conditions throughout the storage period studied (20 weeks). The concentration of phenols in the baked products increased with increasing avocado seed powder. The color of all the queen cake formulations was liked very much by the sensory panel. The aroma of 0% and 15% ASP was liked very much, while the other formulations (30% and 50%) were liked moderately. The taste rating and overall acceptability decreased with an increasing avocado seed powder in the queen cake formulations. Avocado seed extracts can be incorporated to prepare functional beverages and functional baked products that are acceptable by sensory panelists.

## 1. Introduction

Avocado (*Persea americana* Mill.), belonging to the Lauraceae family, is a tropical fruit with an olive green peel and a thick pale yellow pulp rich in oils [1]. This fruit is appreciated for its sensory attributes and the presence of unsaturated fatty acids [2]. It grows well in Kenya and is commonly consumed while ripe or used as fruit salad [3]. Avocado fruit consumption has increased worldwide in recent years, and part of this fruit (skin and seeds) is discarded during processing and consumption [4]. Previous studies on phytochemical composition have shown that avocado seeds exhibit anti-inflammatory, antihypertensive, and antimicrobial medicinal properties [5, 6].

Studies have also revealed that the avocado seed is rich in nutrients, including vitamins, fat, minerals, proteins, carbohydrates, and bioactive compounds [3, 7, 8].

The seed and peel of three avocado varieties, including Shepard, Hass, and Fuerte, have been reported to exhibit antimicrobial activity against yeast, gram-negative, and gram-positive bacteria [9]. The seeds have been shown to contain the highest amount of antioxidants compared to many fruit varieties and vegetables [10]. Gómez et al. [4] showed that a lyophilized extract from the avocado seed gave good protection against oxidation of fat from meat burgers and oil in water emulsions. Similarly, Rodríguez-Carpena et al. [11] found that the avocado seed extracts had good protection against protein oxidation in fresh meat and had an intense antioxidant activity against lipid oxidation in vitro for meat patties. The antioxidant activities of the extracts are attributed to the high concentration of polyphenols in both the peel and the seed [11].

The avocado seed extracts have been reported to exhibit medicinal properties including reducing blood pressure in hypertensive rats [12]. The avocado seed has been used traditionally to treat hypertension, mycoses, parasitic infections, and also local anesthetic effects that decrease muscle pain [12]. Dabas et al. [13] reported the antihypertensive, hypoglycemic, anticancer, and cholesterol-reducing tendency of *Persea americana* seed extracts, showing the potential use of avocado seeds in the formulation of animal feeds and human food. Avocado-soybean unsaponifiables have been shown to be effective in treating symptomatic knee osteoarthritis [14]. These unsaponifiables have also been shown to inhibit clycooxygenase-2 expression and prostaglandin E2 production in chondrocytes which might have an underlying mechanism of action in osteoarthritis [14].

However, with the various health benefits related to avocado seeds, some antinutritional components have been reported in the seeds. These antinutrients include phytic acid, oxalate, and tannins which are known to reduce protein metabolism and nutrient absorption [1]. Some of the negative effects of these components such as reducing nutrient absorption could be potentially utilized to prepare dietary foods [15]. For instance, phytate has been shown to cause a reduced rate of starch digestion, lowering the glycemic index of foods and thus preventing instances of high blood sugar [16]. Phytic acid has also been reported to lower cholesterol absorption thereby reducing blood cholesterol levels [17]. Further, the antinutritional components could be removed or reduced significantly from the seeds by physical factors such as soaking for 24 hours or boiling for 25 minutes in water [3, 18]. Some antinutrients (tannins and flavonoids) can be removed by drying the seeds prior to processing [19].

Despite the nutritional, antimicrobial, and medicinal properties, avocado seeds are discarded after consuming the fruit. The discarded seeds in garbage heaps attract insects and rodents which are environmental nuisances.

The objective of this study was to utilize avocado seed to develop model functional beverages and functional pastries, determine the optimum time-temperature combination for maximum bioactive component extraction from the avocado seed powder and determine their shelf life, conduct proximate analysis of the seed powder, and conduct sensory analysis of queen cakes containing avocado seed powder.

## 2. Materials and Methods

Unripe Hass varieties of avocados were bought from a farmer who is a member of the Abogeta West Avocado Cooperative Society, Meru County, in Kenya. The fruits were transported to the food science laboratories in Meru University of Science and Technology and allowed to ripen for 10 days in a room at ambient temperatures before use.

### 2.1. Avocado Seed Flour Preparation

The seeds were separated from the pulp and then washed. The clean seeds were grated and air-dried at ambient temperature to a constant weight. The dry grated seeds were pulverized into powder using a warring blender (Ramtons, RM/259, Beijing, China). The powder was sieved using screen number 180 *μ*m. The powder was kept in hermetically sealed containers for further analysis.

### 2.2. Moisture Content Determination

The moisture content of the powder was determined by the oven drying method (AOAC 934.01, 2012) using the conventional oven (Ctra Km: 585.1, Barcelona, Spain). A 2 g avocado seed powder sample was weighed into conditioned and preweighed moisture dishes, and the samples were then dried to a constant weight at 105°C.

(1) $$\begin{array}{c} \text{Moisture }\left(\%\right)=\frac{\text{weight loss }}{\text{weight of sample }}\times 100. \end{array}$$

### 2.3. Crude Protein Content Determination

The crude protein content of the avocado seed powder was determined using the Kjeldahl method (AOAC 988.05, 2012). The amount of protein was calculated from the nitrogen concentration in the sample using 6.25 as the conversion factor [20].

### 2.4. Crude Fat Content Determination

The crude fat content was analyzed by the semicontinuous solvent extraction Soxhlet method (AOAC 2003.06, 2012). The avocado seed flour was transferred into porous thimble that was placed in the extraction chamber suspended above flasks containing petroleum ether. Upon heating the flasks, the petroleum ether evaporated and build up in the extraction chamber and then fell back to the flasks upon extracting the fats. The fat that remained in the flasks was weighed and expressed as percentage fat.

### 2.5. Ash Content Determination

Ash content was determined according to AOAC 923.03, 2012. A 2-gram sample was weighed onto conditioned and preweighed porcelain crucibles. The samples were then incinerated in the muffle furnace (Ceramic Fiber Muffle Furnace, SX3-5-12, Huanghua, China) set at 550°C for 4 hours, cooled in the desiccator, and weighed.

(2) $$\begin{array}{c} \text{Ash}\left(\%\right)=\frac{\text{weight of ash }}{\text{weight of sample }}\times 100. \end{array}$$

### 2.6. Crude Fiber Content Determination

The crude fiber content of the avocado seed powder was analyzed by the Henneberg-Stohmann method [21]. Fat was extracted from the 2 g sample using the Soxhlet method and then transferred to a 500 ml flask where it was boiled under reflux in 200 ml 1.25% sulphuric acid, followed by rinsing with distilled water. The sample was then boiled under reflux in 200 ml 1.25% sodium hydroxide. The insoluble matter was then washed with boiled distilled water and then 1% hydrochloric acid. The insoluble matter was then transferred to porcelain crucibles, dried in the oven, and weighed (W1). The residue was then incinerated in the muffle furnace at 500°C for 4 hours. The crucibles were then cooled, and the weight was recorded as W2. The crude fiber content was calculated as shown below:

(3) $$\begin{array}{c} \text{Crude fiber}\left(\%\right)=\frac{W1-W2}{\text{sample weight}}\times 100. \end{array}$$

### 2.7. Total Carbohydrate Determination

Total carbohydrates were determined by calculating the percentage remaining after all the other components in the avocado seed powder were quantified.

### 2.8. Preparation of Seed Extracts

A 1 g sample of the seed powder was steeped in 100 ml distilled water in sample bottles. The lid was closed, and the samples were heated at 40, 50, 60, 70, and 80°C for a number of period points (5, 10, 15, 20, 25, and 30 minutes) at each temperature, respectively. After heating, the extracts were cooled in ice and filtered using the Whatman No. 1 filter paper under vacuum. The filtrate was then stored at -18°C before analysis. Thereafter, the filtrate was used to determine the total phenol content.

### 2.9. Total Phenolic Content Determination in the Extract

The total phenolic content of the extracts was quantified by the Folin-Ciocalteu method [22]. A standard curve was prepared using gallic acid at 50 mg/l, 100 mg/l, 150 mg/l, 250 mg/l, 350 mg/l, 450 mg/l, and 500 mg/l concentrations. A 0.2 ml seed extract/gallic acid standard was mixed with 0.5 ml of Folin-Ciocalteu reagent, 1.5 ml of 20% sodium carbonate solution, and 7.8 ml of distilled water. The samples were then left to react for 2 hours at 25°C, and subsequently, the absorbance was read at 760 nm on a UV-visible spectrophotometer (UV-VIS spectrophotometer, PD-3000UV, Saitama, Japan). The total phenolic content was expressed as milligrams of gallic acid equivalent (mg GAE)/g dry weight of the powder.

### 2.10. Determination of the Shelf Life of Avocado Seed Powder Total Phenols

The dried avocado seed powder was placed in transparent and amber-colored bottles and stored at 25°C. The samples in transparent and amber bottles were stored in ambient light conditions, while another set of samples in amber-colored bottles was stored in the dark. Sampling was carried out on a monthly basis for six months to determine the stability of the polyphenolic constituents.

### 2.11. Preparation of Model Beverages

The model beverages were prepared by steeping the avocado seed powder in water and adjusting to different pH levels. The avocado seed extract had a pH of 5.5. The pH values of the extracts were adjusted to different levels using various concentrations of citric acid and sodium citrate to obtain model beverages with pH levels commonly encountered in food systems (2.8, 3.8, 4.8, and 6.0). The pH was monitored using HANNA instrument pH meter (HI98129, Bucharest, Romania). Benzoic acid was used as a preservative to minimize microbial degradation of the model beverages during storage. The beverages were divided into two aliquots and kept at refrigerated temperature (2°C) and ambient temperature (25°C), respectively. The residual total phenolic content in the samples was then analyzed during storage time.

### 2.12. Baked Product Preparation

The queen cakes/cupcakes were prepared following the formulations as shown in Table 1 with the addition of four different concentrations of Hass avocado seed powder (0, 15, 30, or 50%).

The ingredients were mixed in the bowl mixer (bowl mixer, 22HI-B, Nottingham, United Kingdom); first, margarine, eggs, and sugar were mixed at low speed for 5 minutes; wheat flour, avocado seed powder, baking powder, salt, and milk were added to the mixture. It was then mixed at a medium speed until it was uniform in consistency. The uniform dough was then placed in cups and thereafter baking tins and baked in the baking oven (commercial bakery oven, PFMT-D90, Chennai, India) set at 175°C for 45 minutes. The queen cakes were then cooled, wrapped in plastic bags, and stored at room temperature for further analysis.

### 2.13. Determination of Total Phenols in the Baked Products

The total phenolic content of the baked products was determined by the Folin-Ciocalteu method [22]. A 1 g sample of the baked product was steeped in distilled water maintained at 80°C for 15 minutes and then filtered under vacuum to attain the extract. A 0.2 ml sample of the extract was mixed with 0.5 ml of Folin-Ciocalteu reagent, 1.5 ml of 20% sodium carbonate solution, and 7.8 ml of distilled water. The samples were then left to react for 2 hours at room temperature, and subsequently, the absorbance was read at 760 nm on a UV-visible spectrophotometer (UV-VIS spectrophotometer, PD-3000UV, Saitama, Japan).

### 2.14. Sensory Analysis of the Baked Products

The queen cakes were evaluated for organoleptic properties including texture, aroma, color, taste, and overall acceptability on a nine-point hedonic scale. A total of 56 untrained panelists (32 men and 24 females) aged between 18 and 50 evaluated the samples. The samples were labeled using a three-digit numeral before being presented to the panelists. The nine-point hedonic scale used to evaluate color, aroma, taste and overall acceptability had a verbal intepretation where 9 denoted like extremely, 5 denoted neither like nor dislike and 1 denoted dislike extremely. For the texture parameter, 9 denotes extremely smooth, 5 denotes neither smooth nor rough, and 1 denotes extremely rough.

### 2.15. Data Analysis

Experiments were carried out in triplicate, and analysis was done using IBM SPSS software (SPSS 25.0 for Windows, SPSS Inc.). The statistical analysis was done by ANOVA using descriptive statistics, post hoc, and the least significant difference (LSD) at 95% confidence level. The means were considered to be significantly different if *P* ≤ 0.05.

## 3. Results and Discussion

### 3.1. Proximate Composition of Hass Avocado Seed Powder

The proximate analyses of the avocado seed powder for moisture, ash, crude protein, crude fiber, crude fat, and total carbohydrates were 14.19, 1.82, 7.05, 4.00, 13.64, and 59.30%, respectively. This study showed that the seed powder had a moisture content of 14.19 ± 0.05 percent, which was lower than 15.10 ± 0.14 percent as reported by Ejiofor et al. [1] but slightly above 14.05 percent as reported by Mahawan et al. [23]. High moisture in flour favours insect infestation [24] and mold growth that causes deterioration [25]. The moisture content of the Hass variety avocado seed falls within the standard moisture content range for flours, which is between 13 to 15 percent [23].

The ash content of the avocado seed flour was 1.82 ± 0.05 and is higher than both maize and wheat flour that ranges between 0.29 and 1.21% as reported by Ntuli et al. [26]. The ash content of the avocado seed powder in this study is higher than 0.84 ± 0.02 as reported by [27]. Egbuonu et al. [28] reported an ash content of 3.82 ± 0.00 that is above the recent findings. Different ecological zones and soils may have caused the differences.

The crude protein content of the avocado seed flour was 7.05 ± 0.48%, which is lower than 15.55 ± 0.36% as reported by [1]. This difference may be attributed to the variety and different ecological zones.

The fat content of the Hass avocado obtained was 13.64 ± 0.48 percent, which was lower than 17.90 ± 0.4 percent reported by Ejiofor et al. [1]. The crude fiber content of the avocado seed was 4.0 ± 0.1, indicating that it can be a good source of fiber. The seed flour has a high carbohydrate content, which is 59.3 percent, showing that it can be a good energy source.

### 3.2. Extraction of Total Phenols

In order to compute the total phenolic content, a standard curve was plotted using gallic acid as the standard with the equation *Y* = 0.0038*X* + 0.0349 and *R*^2^ = 0.9978.

**Figure 1:**
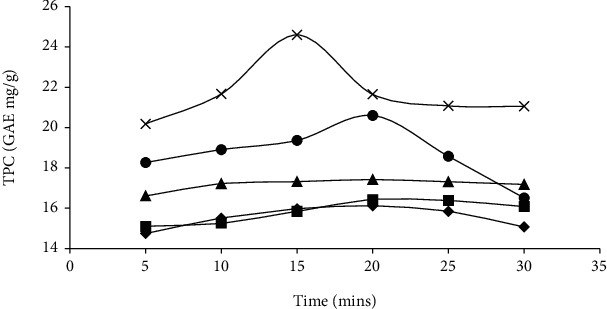
Total phenol content (TPC) of Hass avocado seed powder extracted at different temperatures 40°C (black diamond suit), 50°C (black square), 60°C (black up-pointing triangle), 70°C (black circle), and 80°C (multiplication sign) and time 5 to 30 minutes.

As the extraction temperature increased from 40, 50, 60, 70, to 80°C, it reduced the time to reach the maximum total phenol content (Figure 1).

However, at lower temperatures (40, 50, and 60°C), the extraction of the total phenol content increased gradually without reaching a maximum point after a 30-minute steeping period. On the other hand, extractions at 70 and 80°C reached a maximum point of total phenol content after 20 and 15 minutes, respectively. This result showed that at higher temperatures, there is better extraction of the total phenols within a shorter steeping period. The decrease in the total phenol content after attaining the optimum could be due to the degradation of temperature-sensitive phenols [29]. The increase in extraction of the TPC with an increase in extraction temperature from 40°C to 80°C may be attributed to the higher temperature causing the breakdown of the plant's cellular components, hence releasing bound phenolic compounds [30]. The temperature rise also increases phenols' solubility, leading to higher extraction [31]. The highest total phenol (24.59 ± 0.14 mg/g GAE) was extracted at 80°C for 15 minutes. According to findings reported by Mazyan et al. [32], the highest TPC extracted from avocado fruit flesh was recorded at 105°C and reduced with increased extraction time from 10 to 30 minutes. This shows that total phenolic compounds degrade at high temperatures when exposed for long periods. Further, in a subcritical water extraction process, the optimum TPC was obtained at an extraction temperature of 200°C for 20 minutes [33]. Similarly, Herrero et al. [34] reported that rosemary (*Rosmarinus officinalis*) had the highest antioxidant activity when extracted at 200°C for a short period. Therefore, extraction time plays a vital role at higher temperatures in ensuring minimum degradation of the bioactive compounds.

### 3.3. Shelf Life of Total Phenolic Compounds Extracted from Avocado Seed Powder

The stability of the total phenols in Hass variety avocado seed powder during storage for 6 months at room temperature, under three different storage conditions, is shown in Table 2.

The results show that at all storage conditions, the phenol contents reduced with storage time up to 6 months. However, there was no significant difference (*P* > 0.05) in total phenol content among the three treatments at any storage period. For each treatment, the decrease in the total phenol content during storage was significantly different (*P* < 0.05) after two months of storage and continued to decrease up to 6 months. After 6 months of storage, the percentage retention of the total phenols was approximately 76.38, 69.37, and 65.76% in samples stored in darkness, in amber bottle, and in transparent bottle, respectively. This showed that light influenced the rate of phenol degradation, which agrees with the study conducted on dried *Piper betle* L. extracts [35].

### 3.4. Model Beverages

**Figure 2:**
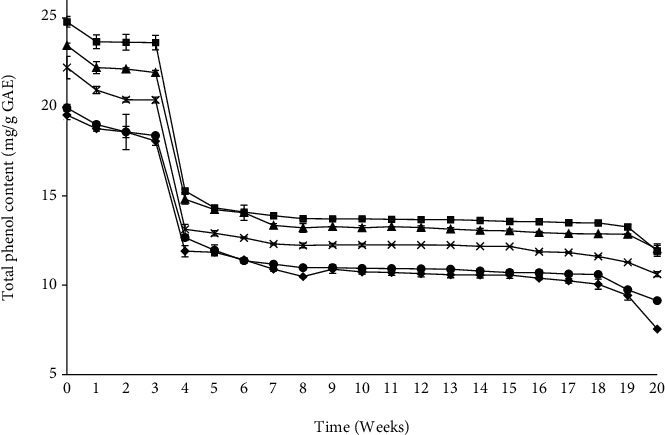
Effect of pH 2.8 (black diamond suit), 3.8 (black circle), 4.8 (multiplication sign), 5.5 (black square), and 6.0 (black up-pointing triangle) on the total phenol content of refrigerated (2°C) model beverages for 20 weeks.

**Figure 3:**
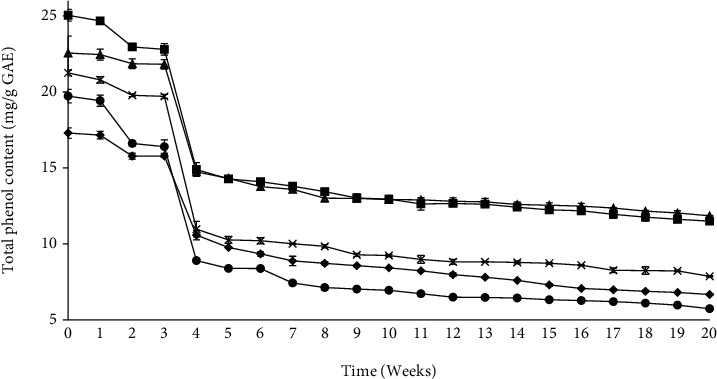
Effect of pH 2.8 (black diamond suit), 3.8 (black circle), 4.8 (multiplication sign), 5.5 (black square), and 6.0 (black up-pointing triangle) on the total phenol content of model beverages stored under ambient (25°C) temperatures for 20 weeks.

The total phenol content was lower in model beverages with lower pH levels (2.8, 3.8, and 4.8) compared to those at higher pH levels (5.5 and 6.0) stored under refrigerated (2°C) conditions (Figure 2) or ambient conditions (25°C) (Figure 3) for 20 weeks. Throughout the storage period, the model beverage with pH 5.5 recorded the highest total phenol content while that with pH 2.8 had the lowest total phenol content.

Akbal and Onar [36] study on the decomposition of various phenol solutions found that high pH levels promote the formation of carbonate ions, which are scavengers of the hydroxyl ions, which reduces the degradation of phenols. It was noted that at pH 6.0, the total phenol content reduced; this is in line with the findings by Friedman and Jürgens [37] that phenolic compounds are damaged when they are exposed to high pH values. In this study, it was noted in particular that pH 5.5 is the best pH value for the beverages since most phenols were retained over the storage time.

After 21 days of storage, the total phenol content of the model beverages dropped rapidly. The degradation of the phenols may be due to the formation of chalcones through hydrolytic reactions which are then rapidly converted to phenolic acids [38]. This is in line with the findings by Diaconeasa [38] that showed a rapid drop of the phenol content of chokeberry and blackcurrant jam.

Storage temperature affected the total phenol content of the model beverages differently. The total phenol content of model beverages at high pH levels was not different after 20 weeks of storage, irrespective of whether they were stored under refrigerated or ambient conditions. However, model beverages at lower pH values and stored at room temperature lost more phenols than those stored under refrigeration. This is in line with the findings by [39], who noted that the phenolic compounds are highly unstable at room temperature or higher. The degradation of the polyphenols in the model beverages is related to their isomerization, enzyme hydrolysis, and oxidation that may have been favoured by the storage conditions [40]. In general, comparing the trend of changes in the TPC in relation to the storage temperatures, the higher the storage temperature, the higher the rate of the total phenol loss was across the storage period. Therefore, storage at lower temperatures (2°C) leads to the retention of more phenols in the beverages over the storage period.

### 3.5. Determination of the Total Phenol Content in the Baked Products

**Figure 4:**
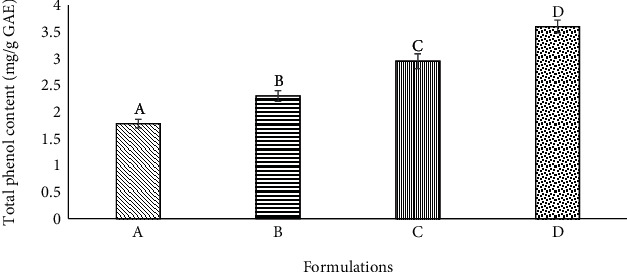
The total phenol content of the queen cakes with different levels of avocado seed powder in formulations A (0%), B (15%), C (30%), and D (50%).

The total phenol content for the baked product (queen cakes) increased with increasing avocado seed powder in the formulation (Figure 4).

The total phenol content of the baked products (queen cakes) increased with the increase in avocado seed powder as a result of the increase in avocado seed powder content which carried the total phenols. Comparing the total phenol content of the avocado seed powder with that of baked product, it is observed that the baked products lost approximately 85% of the total phenols. This may be attributed to high baking temperatures which may have denatured most of the phenolic compounds [29]. High processing temperatures have also been shown to reduce the total phenol content of strawberries [41].

### 3.6. Sensory Analysis of Baked Products (Queen Cakes)

**Figure 5:**
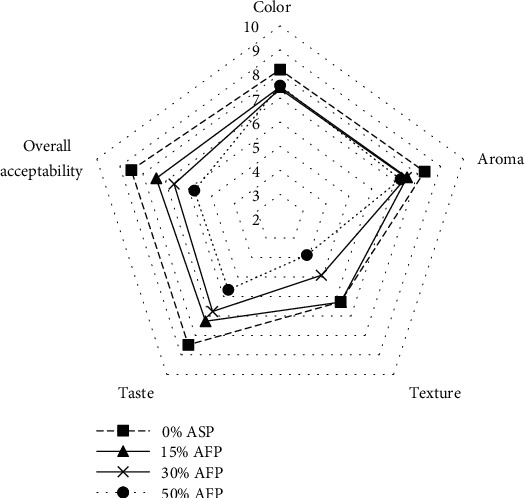
Hedonic scale rating on the acceptability of sensory parameters of the queen cakes containing 0 (black square), 15 (black up-pointing triangle), 30 (multiplication sign), and 50 (black circle) % avocado seed powder (AFP).

As the avocado seed powder increased in the baked product formulation, the rating of texture, taste, and overall acceptability decreased while those of color and aroma did not change significantly (Figure 5).

For all the organoleptic properties of the queen cakes tested by the panelists, there were no significant differences (*P* > 0.05) observed on the color, aroma, texture, taste, and overall acceptability. In terms of color, the 0% queen cake formulation had the highest weighted mean of 8.18 ± 1.77 with a verbal interpretation of like very much. The color acceptability mean for the other formulations containing avocado seed powder was 7.46 ± 1.3, 7.38 ± 1.4, and 7.52 ± 1.3 for 15, 30, and 50%, respectively. The mean for the aroma for all the formulations was not significantly different; formulations with 0% ASP and 15% ASP had a verbal translation of like very much, and on the other hand, the aroma for 30% and 50% ASP was liked moderately.

The taste of the queen cakes containing avocado seed powder was not significantly different from the 0% ASP formulation. The acceptability of the taste reduced with an increase in avocado seed powder, which is attributed to the distinctive aftertaste that increased with increase of the ASP in the formulations [23].

In terms of texture, the queen cakes with the 0% ASP had a mean of 6.27 ± 1.95 with a verbal translation of slightly smooth; the other formulations had a mean of 6.29 ± 1.88 (15%), 4.91 ± 1.79 (30%), and 3.88 ± 2.05 (50%) with a verbal interpretation of slightly smooth, neither rough nor smooth, and slightly rough, respectively. The roughness of the cakes increased with the increase in avocado seed powder in the formulation.

## 4. Conclusion

Based on the findings in this present study, avocado seed powder phenols are more stable when they are stored in darkness at room temperature since they retain 76.38% after six months. The seed extracts can be used to fortify beverages in order to improve the phenolic content of the product. The storage temperature and pH of the model beverage extracts influenced the degradation of the phenols, with pH 5.5 exhibiting high stability at both storage temperatures. Hass avocado seed extract can prepare concentrates for incorporation into beverages that are stable at all pH levels for 21 days. The baked products had a higher phenol content than the control (0% ASP), and the queen cakes produced using 15% avocado seed powder had acceptable organoleptic properties that were comparable with the queen cakes with 0% avocado seed powder, making it the ideal formulation to produce acceptable queen cakes.

## Figures and Tables

**Table 1:** The queen cake formulation with four different proportions of wheat and Hass avocado seed powder.

Ingredients	Formulations			
	A (0%)	B (15%)	C (30%)	D (50%)
Wheat flour (g)	1500	1275	1050	750
Hass avocado seed flour (g)	0	225	450	750
Sugar (g)	800	800	800	800
Baking powder (g)	40	40	40	40
Margarine (g)	800	800	800	800
Eggs	17	17	17	17
Milk (ml)	500	500	500	500
Salt (g)	5	5	5	5

**Table 2:** Changes in avocado seed powder total phenol content (mg/g GAE) during storage for 6 months stored in darkness or in ambient light while in amber bottle and transparent bottle.

Time	Storage condition		
	In the dark	Amber bottle	Transparent bottle
Initial	24.35 ± 0.53^a^	24.49 ± 0.36^a^	24.30 ± 0.17^a^
Month 1	24.29 ± 0.61^a^	24.45 ± 0.29^a^	24.29 ± 0.08^a^
Month 2	23.55 ± 0.1^b^	21.26 ± 0.18^b^	21.15 ± 0.20^b^
Month 3	20.39 ± 0.8^bc^	20.79 ± 0.17^bc^	21.03 ± 0.56^bc^
Month 4	19.38 ± 0.37^c^	19.58 ± 0.18^c^	19.71 ± 0.19^c^
Month 5	18.87 ± 0.43^d^	17.63 ± 0.30^d^	16.93 ± 0.03^d^
Month 6	18.6 ± 0.10^d^	16.99 ± 0.13^d^	15.98 ± 0.13^d^

The values are means ± standard deviation. Means along columns with different superscript letters are significantly different (*P* < 0.05).

## Data Availability

Data are available from the authors, official company websites, and various uniform resource locators (URLs), as listed in References. The data that support the findings of this study are available from the corresponding author upon request.
